# A whole genome analyses of genetic variants in two Kelantan Malay individuals

**DOI:** 10.1186/s11568-014-0004-0

**Published:** 2014-10-21

**Authors:** Wan Khairunnisa Wan Juhari, Nur Aida Md Tamrin, Mohd Hanif Ridzuan Mat Daud, Hatin Wan Isa, Nurfazreen Mohd Nasir, Sathiya Maran, Nur Shafawati Abdul Rajab, Khairul Bariah Ahmad Amin Noordin, Nik Norliza Nik Hassan, Rick Tearle, Rozaimi Razali, Amir Feisal Merican, Bin Alwi Zilfalil

**Affiliations:** 1 Department of Pediatrics, School of Medical Sciences, Universiti Sains Malaysia, 16150 Kubang Kerian, Kelantan, Malaysia; 2 Faculty of Resource Science and Technology Universiti Malaysia Sarawak, Sarawak, Malaysia; 3 School of Dental Sciences, Universiti Sains Malaysia, Kelantan, Malaysia; 4 School of Health Sciences, Universiti Sains Malaysia, Kelantan, Malaysia; 5 Complete Genomics Inc, 2071 Stierlin Court, Mountain View, CA 94043 USA; 6 Sengenics Sdn Bhd, Kuala Lumpur, Malaysia; 7 Centre of Research for Computational Sciences and Informatics in Biology, Bioindustry, Environment, Agriculture and Healthcare (CRYSTAL), Kuala Lumpur, Malaysia; 8 Institute of Biological Science, Faculty of Science, Universiti Malaya, Kuala Lumpur, Malaysia; 9 Human Genome Center, School of Medical Sciences, Universiti Sains Malaysia, Universiti Sains Malaysia, Kelantan, Malaysia

**Keywords:** Malays, Single nucleotide polymorphisms, *Helicobacter pylori*, Beta-thalassemia, Whole genome analysis

## Abstract

**Background:**

The sequencing of two members of the Royal Kelantan Malay family genomes will provide insights on the Kelantan Malay whole genome sequences. The two Kelantan Malay genomes were analyzed for the SNP markers associated with thalassemia and *Helicobacter pylori* infection. *Helicobacter pylori* infection was reported to be low prevalence in the north-east as compared to the west coast of the Peninsular Malaysia and beta-thalassemia was known to be one of the most common inherited and genetic disorder in Malaysia.

**Result:**

By combining SNP information from literatures, GWAS study and NCBI ClinVar, 18 unique SNPs were selected for further analysis. From these 18 SNPs, 10 SNPs came from previous study of *Helicobacter pylori* infection among Malay patients, 6 SNPs were from NCBI ClinVar and 2 SNPs from GWAS studies. The analysis reveals that both Royal Kelantan Malay genomes shared all the 10 SNPs identified by Maran (Single Nucleotide Polymorphims (SNPs) genotypic profiling of Malay patients with and without Helicobacter pylori infection in Kelantan, 2011) and one SNP from GWAS study. In addition, the analysis also reveals that both Royal Kelantan Malay genomes shared 3 SNP markers; HBG1 (rs1061234), HBB (rs1609812) and BCL11A (rs766432) where all three markers were associated with beta-thalassemia.

**Conclusions:**

Our findings suggest that the Royal Kelantan Malays carry the SNPs which are associated with protection to *Helicobacter pylori* infection. In addition they also carry SNPs which are associated with beta-thalassemia. These findings are in line with the findings by other researchers who conducted studies on thalassemia and *Helicobacter pylori* infection in the non-royal Malay population.

## Background

The Malays *(Melayu)* are found across wide areas in the world including Peninsular Malaysia, Borneo (Sabah & Sarawak), Indonesia, Singapore, Brunei, Thailand, Sri Lanka and some of the Cape Malay community in Cape Town, Africa. Among these countries, Malaysia and Brunei formed the majority of the Malay population. In Peninsular Malaysia, the Malays consist of several sub-ethnics groups originating from different ancestral lineages based on their migration many years ago (Paul, 1961). The Malays in Peninsular Malaysia comprises the western Malays (*Melayu Minang*), southern Malays (*Melayu Jawa* and *Melayu Bugis*) and northern Malays (*Melayu Kelantan* and *Melayu Kedah*) according to their settlements in the Peninsular Malaysia.

The SNP genotyping data previously reported by Hatin et al. (2011) has placed the Kelantan Malay or *Melayu Kelantan* as an outlier to the other Malay ethnic groups in the Peninsular Malaysia. Kelantan Malay (*Melayu Kelantan*) has an ancestry that is more divergent than other Malay populations due to their historical links and geographical location at the northern part of the Peninsular Malaysia. Meanwhile, other Malays from the western and southern regions of the Peninsular Malaysia have more historical and cultural links with respective populations from the Indonesian archipelago, whereas Kelantan Malay (*Melayu Kelantan*) shows limited links with these populations (Hatin et al., 2011). The uniqueness shown by the Kelantan Malay (*Melayu Kelantan*) has sparked interests in understanding the Malaysian population in Malaysia in particular the Malay sub-ethnic groups.

The sequencing of two members of the Royal Kelantan family genomes will provide insights on the Kelantan Malay (*Melayu Kelantan*) whole genome sequences. The two Royal family members are the descendents of the Sultan Muhammad IV, the ruler of the Kelantan state from 1911 to 1920. The sequence will allow the construction of a Kelantan Malay (*Melayu Kelantan*) reference genome, the identification of variants specific to the Kelantan Malay (*Melayu Kelantan*) ethnic group and the positioning of Kelantan Malay (*Melayu Kelantan*) in the broad prehistory of both the Malay Peninsula and the Southeast Asia in general.

The whole genome sequence of the Royal Kelantan Malay individuals should reveal the genetic variants associated with *Helicobacter pylori* infection and thalassemia in Kelantan Malay (*Melayu Kelantan*) population. Although the Malays are the predominant ethnic groups in Malaysia, a study has reported the low prevalence of *Helicobacter pylori* infection in the Malays compared to the Chinese and the Indians (Goh, 2009). The high prevalence of *Helicobacter pylori* infection in the Chinese and the Indians might be due to the high prevalence from the country they originated from, which are the Southern China and the Southern India (Goh, 2009). The *Helicobacter pylori* prevalence also varied from a low range of 26.4% in Kota Bharu in the north east of the Peninsular Malaysia to a substantially high of 55.0% in Kota Kinabalu in the Sabah state of Borneo (Goh & Parasakthi, 2001). These findings were further supported by the prevalence of *Helicobacter pylori* infection which was reported to be low prevalence in the north-east as compared to the west coast of the Peninsular Malaysia (Goh & Parasakthi, 2001; Uyub et al., 1994). Due to the history of immigration thousand years ago, there are few implications related to the Malays *Helicobacter pylori* prevalence as the Malays isolates shared the same origin as the Indian isolates (Tay et al., 2009). Previous genome wide association study conducted by our group (Maran, 2011) revealed the presence of protective SNPs contributed to the low prevalence of *Helicobacter pylori* infection in Kelantan.

## Results

### SNPs identification

The sequence data was aligned to the NCBI human reference genome (build 37) with an average coverage depth of 40 fold. As both the Royal Kelantan Malay are first cousins, they are expected to share 1/32 of their genomes. In our result we found that 1/30 (93.6 Mb of 2,830 Mb) of the sequence data generated showed similarity for both genomes indicating consistency of our result with their relationship as first cousin.

Based on the sequence that covered K1 and K2 genomes, we identified over 3.4 million SNPs which comprised over 2 million heterozygous SNPs and 1.4 million homozygous SNPs. From the identified SNPs, more than 390,000 SNPs were identified to be novel when compared to NCBI dbSNP (build 132) (Table 1)

**Table 1:** **Summary statistics of SNPs**

**Class**	**Total (K1)**	**Total (K2)**
Known homozygous SNPs	1,454,276	1,441,837
Novel homozygous SNPs	22,028	22,936
Known heterozygous SNPs	2,117,041	2,094,134
Novel heterozygous SNPs	371,391	355,511

### Individual genome comparison

We compared the SNPs present in both K1 and K2 genomes against the NCBI dbSNP database (build 132). The whole genome sequence data of K1 and K2 were transformed into virtual Affymetrix GeneChip Human Mapping 50k *Xba*1 Array to allow the comparison of SNP calling with the genotype data of the four Malay sub-ethnic groups in the peninsular Malaysia (*Melayu Kelantan, Melayu Minang, Melayu Jawa* and *Melayu Bugis*) (as reported by Hatin et al. 2011). Based on the genotyping calls, about 54,794 autosomal SNPs were identified to be similar between the Royal Kelantan Malay genomes and the Kelantan Malay (*Melayu Kelantan*)*.*

We compared the Royal Kelantan Malay genomes with two other individual genomes, the Han Chinese and the South Asian Indian female genome that have been sequenced (Gupta et al., 2012; Wang et al., 2008). The basis of this comparison was to look for any admixture between the genomes as reported in the previous study, where the distinct genetic difference of the Malays was possibly due to the admixture between the Kelantan Malay (*Melayu Kelantan*) with other Indian populations (Hatin et al., 2011). The admixture could possibly occur by Indians who migrated from India in second century AD (Hatin et al. 2011).

The Royal Kelantan genomes were compared with two genomes, the Han Chinese and the South Asian Indian Female (SAIF) from the published personal genomes. The SNPs level comparisons using dbSNP (build 132) showed that this individual shared 98% SNP with Han Chinese and 95% with SAIF. This most probably indicates the Royal Kelantan subjects may have ancestary link with Han Chinese and SAIF.

The whole genome individual sequencing identified an average of over 3 million SNPs per individual. Many studies of other whole genome samples from the Han Chinese (Wang et al., 2008), the South Asian Indian Female (Gupta et al., 2012), the SJK-Korean (Ahn et al., 2009) and the Southeast Asia Malays in Singapore (Wong et al., 2013) have reported the discovery of a consistent number of SNPs. The analysis of the K1 and K2 individual genomes has also revealed a level of well-attested variation similar to other non-African populations. K1 carried 3,946,306 while K2 3,906,477 of such variants, compared to the human reference genome, with the average range of 3,956,074 ± 39,778 variants to a control group of the Caucasian and the Asian genomes based on the Complete Genomics public data. It was found that the overlapping variation between the two Royal Kelantan Malay individuals, K1 and K2 were about 2,542,089 (19.3%) and the unique variants of K1 and K2 were about 1,404,217 (10.7%) and 1,364,388 (10.4%), respectively.

Of the variants shared by both Kelantan Royal genomes, the overlapping variants of both individual genomes are shared with the Asian and the African genomes. With the availability of the Asian genomes for comparison, the Han Chinese and the South Asian Indian, the positioning of Kelantan Malay (*Melayu Kelantan*) genomes were able to be determined.

## Discussion

### Royal Kelantan Malay genomes associated with Helicobacter pylori infection

The two Royal Kelantan genomes were analyzed for the SNP markers associated with *Helicobacter pylori* infection. By combining the SNP information from literatures, GWAS study and NCBI’s ClinVar database, 18 unique SNPs were selected for further analysis. From these 18 SNPs, 10 SNPs came from the previous study of *Helicobacter pylori* infection among the Malay patients (Maran, 2011), 6 SNPs were from NCBI’s ClinVar database (Landrum et al., 2014) and 2 SNPs from the genome wide association studies (GWAS).

The analysis revealed that both Royal Kelantan Malay genomes shared all the 10 SNPs identified by Maran (2011) and one SNP from the GWAS study (Mayerle et al., 2013) (Table 2). The findings could be attributed by a disease which is linked to the survival and reproductive success, making it a strong selective force in human evolution (Myles et al. 2008). Based on recent studies by Lee et al. (2012) and Maran et al. (2013), the low prevalence of *Helicobacter pylori* infection in Malay residing in Kelantan was probably due to the local dietary practice and also genetic factors that were found to be protective against the bacteria.

**Table 2:** **List of all SNPs associated with H. pylori infection in the Royal Kelantan Malay genome**

**dbSNP ID**	**Source**	**Identified**
rs10496563	Study (Maran, 2011)	Yes
rs9319171	Study (Maran, 2011)	Yes
rs1533949	Study (Maran, 2011)	Yes
rs221493	Study (Maran, 2011)	Yes
rs10487849	Study (Maran, 2011)	Yes
rs2120441	Study (Maran, 2011)	Yes
rs1941158	Study (Maran, 2011)	Yes
rs10435337	Study (Maran, 2011)	Yes
rs200032	Study (Maran, 2011)	Yes
rs2896092	Study (Maran, 2011)	Yes
rs397514620	ClinVar database	No
rs45575636	ClinVar database	No
rs121918442	ClinVar database	No
rs58238559	ClinVar database	No
rs387906528	ClinVar database	No
rs72552778	ClinVar database	No
rs10004195	GWAS (Mayerle et al. 2013)	Yes
rs368443	GWAS	No

Interestingly, by using the whole genome sequencing approach, we were able to identify in the two uninfected Royal Kelantan Malay individuals similar SNPs that were observed to be protective towards *Helicobacter pylori* infection in the non-royal Kelantan Malay individuals. The genetic variants that were previously studied by Lee et al. (2012) and Maran et al. (2013) most probably were responsible for the protection against *Helicobacter pylori* infection in the two Royal Kelantan Malay individuals. The findings of these SNPs in the two individuals have thus provided credence to our proposal that the genomes of this two Royal Kelantan Malay individuals be the reference genome sequence for Kelantan Malay (*Melayu Kelantan*).

### Royal Melayu Kelantan genome associated with thalassemia

The two Royal *Melayu Kelantan* genomes were also analyzed for SNP markers associated with thalassemia as thalassemia is a public health problem which is also an inherited disease. It is common in the Malays with 5% carrier rate (George, 2013). 231 SNPs were selected for the analysis. 228 SNPs came from NCBI’s ClinVar database (Landrum et al., 2014) and 3 SNPs from the GWAS studies.

The analysis revealed that both Royal Kelantan Malays *(Melayu Kelantan)* genomes shared 3 SNP markers, where all three markers were associated with beta-thalassemia. The SNPs implicated in the disease, rs1061234, rs1609812 and rs766432 were identified in the HBG1, HBB and BCL11A genes, respectively. BCL11A functions as a myeloid and B-cell proto-oncogene and plays important roles in leukemogenesis and hematopoiesis. An essential factor in lymphopoiesis is required for B-cell formation in fetal liver and may function as a modulator of the transcriptional repression activity of ARP1. It is expressed at high levels in brain, spleen thymus, bone marrow and testis. In addition, it is expressed in CD34-positive myeloid precursor cells, B-cells, monocytes and megakaryocytes. Its expression is tightly regulated during the B-cell development. HBB is involved in oxygen transport from the lung to the various peripheral tissues and as an endogenous inhibitor of enkephalin-degrading enzymes such as DPP3, and also as a selective antagonist of the P2RX3 receptor involved in pain signaling, where these properties implicate it as a regulator of pain and inflammation. HBB is known as a gene associated with beta-thalassemia. The absence of beta chain causes beta (0) -thalassemia while reducing the amounts of detectable beta globin causes beta (+) -thalassemia. In the severe forms of beta-thalassemia, the excess alpha globin chains accumulate in the developing of erythroid precursors in the marrow. Their deposition leads to a vast increase in the erythroid apoptosis, which in turn causes ineffective erythropoiesis and severe microcytic hypochromic anemia. Clinically, beta-thalassemia is divided into thalassemia major which is transfusion dependent, thalassemia intermedia (of intermediate severity) and thalassemia minor that is asymptomatic.

Lastly, HBG1 is normally expressed in the fetal liver, spleen and bone marrow. Two gamma chains together with two alpha chains constitute of fetal hemoglobin (HbF), which is normally replaced by adult hemoglobin (HbA) at birth. With some beta-thalassemias and related conditions, gamma chain production continues into adulthood. The two types of gamma chains differ at residue 136, where glycine is found in the G-gamma product (HBG2) and alanine is found in the A-gamma product (HBG1). The former is predominant at birth.

### Royal Melayu Kelantan genome and pharmacogenomics

Further analysis of the two Royal Melayu Kelantan genomes was performed by analyzing their pharmacogenomics properties. The association between the SNP and the drugs were identified based on the Pharmacogenomics Knowledge Base (PharmGKB) database (http://pharmgkb.org) (Whirl-Carrillo et al. 2012. From over 3.5 million SNPs identified, over 1,200 variants were identified to either affect the toxicity or efficacy of numerous drugs that are available in the market. For example, the variation in the IGF2BP2 (rs4402960) has been reported to enhanced the effect of repaglinide treatment in type 2 diabetes patient in China (Huang et al., 2010).

Additionally, we also identified a variation in the FCER1G gene (rs11587213). This variation may contribute to the development of aspirin-intolerant asthma (AIA) by altering the toxicity level (Palikhe et al., 2008). In 2-23% of adults with asthma, and rarely in children with asthma, aspirin (acetylsalicylic acid) could cause asthma exacerbations (Obase et al., 2005). Within 3 hours of ingestion of aspirin, individuals with aspirin-intolerant asthma (AIA) could develop bronchoconstriction, often accompanied by rhinorrhea, conjunctival irritation, and scarlet flush (Obase et al., 2005). In severe cases, a single therapeutic dose of aspirin can provoke violent bronchospasm, loss of consciousness, and respiratory arrest (Obase et al., 2005). Besides these variations, we also identified other disease associated SNPs found in both Royal individuals (Table 3).

**Table 3:** **Disease associated SNPs with their respective drugs**

**dbSNP ID**	**Gene**	**Disease**	**Drugs**
**rs3129900**	C6orf10	Osteoarthritis and acute pain	Lumiracoxib
**rs4149056**	SLCCO1B		Simvastin
**rs3918290**	DPYD	Purine-Pyrimidine Metabolisme, Inborn errors	Fluorouracil
**rs9923231**	VKORC1	Thrombosis	Warfarin

## Conclusion

Our findings suggest that the Royal Kelantan Malays carry the SNPs which are associated with protection to *Helicobacter pylori* infection. In addition they also carry SNPs which are associated with beta-thalassemia. These findings are in line with the findings by other researchers who conducted studies on thalassemia and *Helicobacter pylori* infection in the non-royal Malay population. This whole genome sequence of Royal Kelantan Malays provides a reference genome for the Kelantan Malays sub-ethnic group and will be useful to those conducting comparative and evolutionary population studies.

## Methods

### Sample collection, library construction and sequencing

Approval from the Universiti Sains Malaysia (USM) Research Ethics Committee was obtained and the written informed consent from the two subjects were taken. A total of 3 ml blood was taken from each of the selected male subjects of the Royal Kelantan family. The two royal individuals were denoted as K1 and K2. Both individuals were not known to have hereditary diseases based on the interview conducted on them. The genomic DNAs were extracted using QIAGEN (Germany) Blood Mini Kit with the final concentration of genomic DNA of more than 100 ng/ul.

The genomic DNAs obtained were used in the preparation of the whole genome libraries according to the Complete Genomics library preparation protocols (Complete Genomics Inc., USA). The sequence data were then obtained by performing two primary components of sequencing technology developed by Complete Genomics Inc., the DNA nanoball arrays, the DNB™ arrays and the combinatorial probe-anchor ligation reads, cPAL™ reads (Complete Genomics Inc., USA).

The SNPs associated with *Helicobacter pylori* were searched from the variant list of both individual genomes according to the list of dbSNP ID that corresponds to the SNP that was previously reported by (Maran, 2011). The functional impact of each SNP was then evaluated using the F-SNP database (http://compbio.cs.queensu.ca/F-SNP/). Searchers on the NCBI Single Nucleotide Polymorphism database (dbSNP) (Sherry et al. 1999), Online Mendelian Inheritance in Man (OMIM) (Amberger et al., 2009) and Genatlas (http://genatlas.medecine.univ-paris5.fr/) were performed to obtain information on the sequence variants.
